# On the Non-Asymptotic Stability of a Directional Gyroscope

**DOI:** 10.3390/s26175465

**Published:** 2026-08-29

**Authors:** Yakov Isaakovich Binder, Daniil Yurievich Larionov, Roman Vadimovich Shalymov

**Affiliations:** Department of Laser Measurement and Navigation Systems, Saint Petersburg Electrotechnical University “LETI”, ul. Professora Popova 5, 197022 St. Petersburg, Russia; j459190@yahoo.com (Y.I.B.); lariondan@yandex.ru (D.Y.L.)

**Keywords:** Euler’s equations, selectivity, directional gyroscope, numerical integration, non-asymptotic stability, hodograph, two-degree-of-freedom balanced gyroscope, meridian line, gyrocompass, free gyroscope

## Abstract

This article examines the behavior of gyroscopic orientation systems, which, unlike inertial navigation systems and gyrocompasses (with internal and external correction), are described solely by Euler’s equations. It is shown that, contrary to established views, obtaining the position of the meridian line using a balanced directional gyroscope corrected by information on the transport angular velocity (or a two-axis inertial platform controlled by integrating gyroscopes) is non-asymptotically stable over a wide range of initial conditions, disturbance torques, and parameters of the object’s motion on the Earth’s surface—more than sufficient for practical use. A comparison of a directional gyroscope and a gyrocompass provides a new interpretation of the role of pendulosity in the design of gyroscopic orientation systems. The fundamental theoretical propositions of the present work are accompanied, to the required extent, by the results of numerical solutions of the equations of motion.

## 1. Introduction

The directional gyroscope, or DG (to avoid cluttering the text, we will use the term “DG” regardless of whether the device includes a correction for angular velocities caused by motion on the Earth’s curved surface or not; historically, the former case was called a “velocity compass”)—also known as the azimuth gyroscope in maritime practice and as the heading indicator in aviation—should be classified as a flight control and navigation system of the previous century. The DG was—and, in some cases, remains, due to the well-known conservatism of military designs—the primary heading device for aircraft, tanks, and mine-and-torpedo armament. The first control devices, the “Vertikant” and “Horizont” (a name sometimes used to denote the artificial horizon, which is incorrect since we are talking about a different device here [1,2]) of the V-2 ballistic missile and its American and Soviet descendants, are conceptually close to the DG.

In the navy, the primary heading indicator was the gyrocompass [3], which, when the Schuler tuning [2,4] was neglected (in other words—in most cases, albeit for various reasons), was subject to perturbations by ship’s acceleration (so-called ballistic deflection [4]). Externally corrected gyrocompasses [5], which saw the light of day in the last quarter of the previous century, became dual-mode, and were switched to the gyro azimuth mode (immune to disturbances) during accelerations and maneuver operations.

In the classic work [6], “gyrocompass … determines the direction of true north by an inertial measurement of the Earth’s daily rotation with the aid of a gravity tracker”, while the (non-selective) DGs “use the properties of a gyroscope to stabilize … external directional information”, and “if the slaving torque compensates for the angular velocity of the reference meridian, with respect to inertial space, that is due to both the vertical component of Earth’s daily rotation and vehicle velocity with respect to Earth, … maintains its reference direction for a long period”. Here is another quote from a respected author specializing in the study of gyro directional instruments, cited in a book by his students: “… DGs differ from gyrocompasses and magnetic compasses in that they lack position-dependent torque that steers the device’s moving part into the meridian plane, and thus qualify as instruments with neutral equilibrium (i.e., balanced) … which explains why DGs allow one to determine not the vehicle’s heading, but only its increments, and … the accuracy of measurements … is ensured only for a limited period of time” [7]. The majority of subsequent publications devoted to azimuth direction indicator devices (DG, gyrocompass, etc.), including modern ones ([5,8]), consider completely different features of their construction and use—including their fusion with other measuring devices—without questioning the interpretations set out in [7,9]. This opinion adequately reflects the views of the scientific community as a whole: a two-degree-of-freedom balanced gyroscope (taking a cue from [6], hereinafter we will refer to the two-degree-of-freedom balanced gyroscope as 2dfbg), controlled by torque generators based on external information only on object’s angular velocity, lacks selectivity to the meridian line; as a result, it requires an external initial directional alignment, the storage error of which, given constant values of the disturbance torques on its axes, will increase over time.

Below, the validity of this statement and some related issues are considered. Based on the expected results and the authors’ existing experience, this paper will cover navigation on the Earth’s surface for marine (surface vessels and submersibles, with displacement and planing hulls, autonomous unmanned vehicles, and other watercraft), ground and burrowing vehicles (remotely operated, towed, self-propelled, as well as wireline logging tools and pipeline in-line inspection systems, regardless of route type) of any purpose. These restrictions do not preclude subsequent partial lifting.

## 2. Basic Concepts

As far as the authors know, a rigorous, specialized definition of “non-asymptotic stability” as a basic concept is not commonly used in dynamical systems theory; instead it is introduced through negation, i.e., the absence of asymptotic stability. This means that the Lyapunov criterion for systems of this kind is satisfied without asymptotic approach to the equilibrium position. Let us consider the bare minimum of the most relevant examples in the context that is of interest to us.

A classic example of non-asymptotic stability is an ideal mathematical pendulum without energy dissipation. Deflected by a certain limited angle, it performs harmonic oscillations around the equilibrium position, remaining within its bounded vicinity yet never reaching said equilibrium.

Another example is a linear controller residing on the stability boundary according to the Nyquist criterion; under small perturbations, it exhibits either continuous oscillations or a constant shift. Finally, a nonlinear Van der Pol oscillator has a stable limit cycle in the steady-state mode, but does not converge to an equilibrium point.

**Figure 1:**
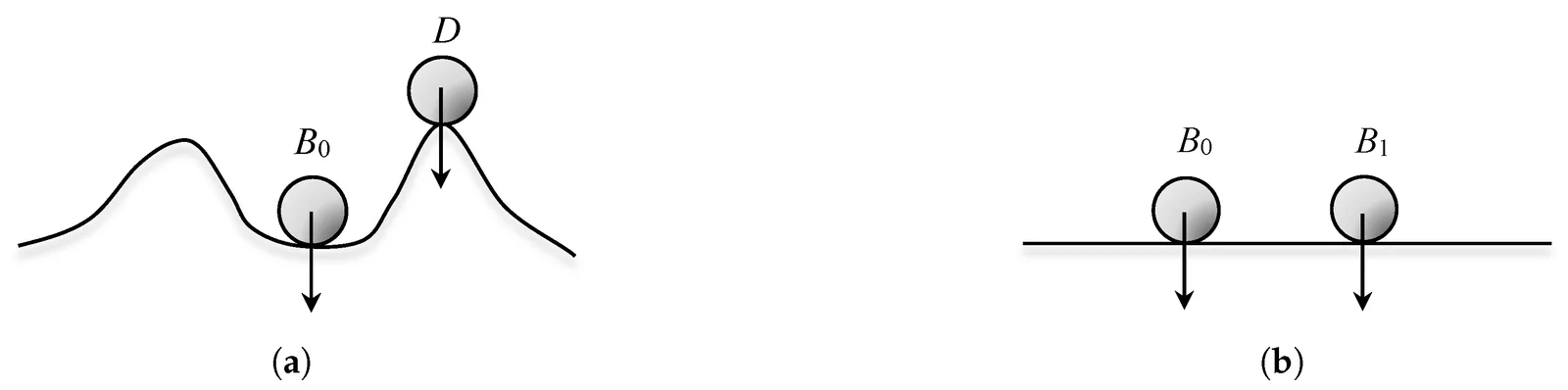
A ball on a surface: (**a**) residing in a stable equilibrium position only until the deviation goes beyond a certain boundary, determined, for example, by the *D* point (having gone beyond this boundary, it will no longer return to the stable equilibrium position $B_{0}$, but will instead move from point *D* to the right); (**b**) in a neutral equilibrium—both $B_{0}$ and $B_{1}$ are equilibrium positions.

As shown below, the motion of a DG is described by two nonlinear inhomogeneous differential equations. In the general case, in order to study such systems (see Figure 1a), the concepts of “global”, “in the large” and “local” stabilities are introduced. In this paper we will be interested only in the latter, which boils down to stating the presence of a stability region without defining its boundaries [10].

An analysis of the stability “in the large” incorporates such definition, and performing it in this case would not present any fundamental difficulties. It would, however, lead to a significant increase in the volume of the article—in the authors’ opinion, absolutely unjustified from a practical perspective: the actual boundaries of the initial deviations [10] for a DG are much narrower than the boundaries of stability.

The concept of “selectivity”, widely used in this paper, is also semantically related to the conceptual apparatus of stability. It implies the existence of a stable equilibrium position for a DG—albeit unattainable due to its non-asymptotic nature—as opposed to neutral one (see Figure 1b).

## 3. Equations of Motion for a Directional Gyroscope and Their Analysis Methodology

**Figure 2:**
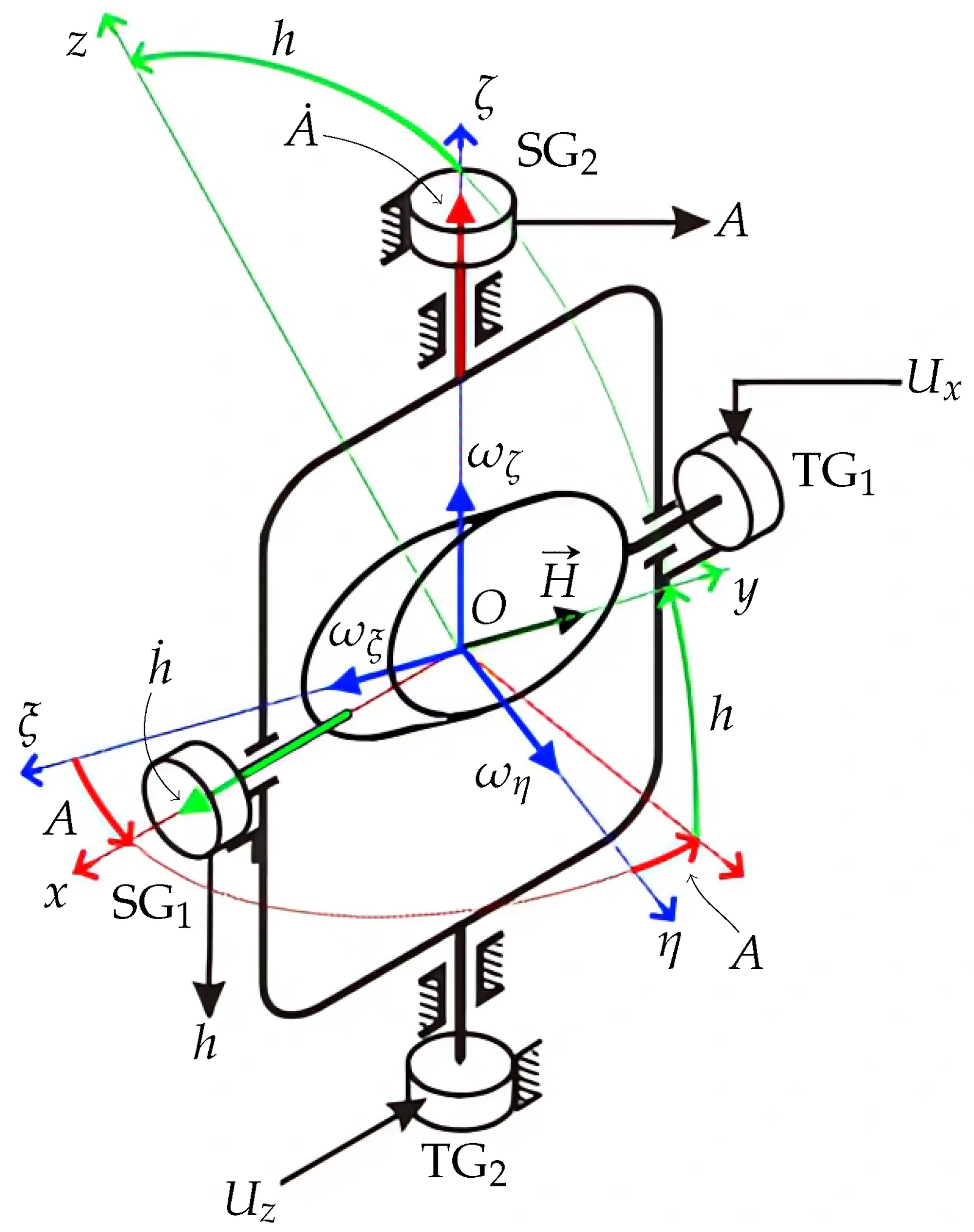
Two-degree-of-freedom balanced gyroscope with signal and torque generators. Orientation of the Résal’s system relative to the reference frame (i.e., geographic coordinate system) is shown. $U_{x,z}$ is compensation for transport angular velocity and systematic wander.

Let us look at Figure 2, which shows a 2dfbg whose outer gimbal is nominally vertical (so-called altazimuth gimbal, hereinafter Alt-Az). The *x*, *y*, and *z* axes are the gyro element-fixed (i.e., Résal’s) coordinate system, where *y* is the spin axis of a gyro with angular momentum *H*, and *x* is the axis of the gyro element gimbal’s rotation relative to the outer gimbal. Signal generators (SGs) and torque generators (TGs) are positioned opposite each other on the axes of the Alt-Az gimbal. According to our problem statement, these generators apply torques that compensate, firstly, for the vehicle’s angular velocities caused by its motion on the Earth’s curved surface as well as Earth’s daily rotation (we will call these angular velocities the *transport velocities*, and these torques the *azimuth correction torques*); and, secondly, for the systematic components of the error torques. The latter—expressed in terms of equivalent input angular rate—are compensated over the course of predetermined calibration with an accuracy determined primarily by the stability of these torques, as well as by the errors of the calibration procedure itself. No other correction signals (pendulosity, orthogonality loop-control, etc.), which are fundamental when functioning on a real vehicle, are introduced here; we only study the DG stability during the vehicle’s uniform motion along an Earth’s rhumb line. To conclude this brief description, let us clarify that the azimuth correction torque is applied by ${\mathrm{TG}}_{1}$ about the axis of the gyro element gimbal, causing the gyroscope to precess around the axis of the outer gimbal. In turn, torque generator ${\mathrm{TG}}_{2}$, installed on this axis, provides the horizontal correction about the axis of the gyro element gimbal. Rotation angles about the axes of the gyro element gimbal (*h*) and outer gimbal (*A*) are acquired from signal generators ${\mathrm{SG}}_{1}$ and ${\mathrm{SG}}_{2}$.

Let us remark here that the diagram in Figure 2 can also describe a double-gimbal inertial platform using more modern, precision gyroscopes than a classic two-degree-of-freedom balanced gyro: liquid-floated gyros, dynamically tuned gyros, gyros with a ball rotor and gas-dynamic or active magnetic bearing—used in integrating gyro (IG) mode. There is no need to depict this diagram again—it is sufficient to mentally replace the 2dfbg rotor installed in the Alt-Az gimbal with a platform with a two-degree-of-freedom (or two single-degree-of-freedom) IGs, whose sensitive axes lie within the plane of the gyro element gimbal such that one of them coincides with its axis, and the other forms a right-hand triple with it and the gyro spin angular momentum vector (directed in the same way as for the 2dfbg in Figure 2). In this case, ${\mathrm{TG}}_{1}$ and ${\mathrm{TG}}_{2}$ operate as stabilizing motors controlled by the signal generators measuring misalignment between the IG’s gyro element and its case. This, for example, is the design of a dual-mode externally corrected DG/gyrocompass [5]. The fundamental behavioral patterns of a 2dfbg and an inertial platform in an Alt-Az gimbal are, in general terms and in most cases of interest to us, obviously similar. In the following discussion, in order to maintain the identity of the objects analyzed in this paper and in the seminal works that we are debating with, we will limit ourselves to considering the 2dfbg, while pointing out the peculiarities introduced by the use of inertial platforms where necessary.

To describe the motion of the DG under consideration, we will orient the *x*, *y*, *z* axes of the Résal’s system, choosing the geographic coordinate system (GCS) Oξηζ as reference frame with the origin *O* coinciding with the gyro’s center of mass (with the gyro itself located at latitude φ and arbitrary longitude). The ξ and η axes lie within the plane of the horizon, with ξ directed tangentially to the circle of latitude to the east and η directed tangentially to the meridian to the north (i.e., along the meridian line). The ζ axis is directed vertically toward the zenith. The GCS has an absolute angular velocity due to the Earth’s rotation and the object’s motion along its spherical surface (approximating the Earth’s surface as a sphere does not impair the correctness of the presented analysis): $\omega_{\xi }=-\frac{V_{N}}{R}$, $\omega_{\eta }=\Omega \cos\varphi +\frac{V_{E}}{R}$, $\omega_{\zeta }=\Omega \sin\varphi +\frac{V_{E}}{R}\tan\varphi$, where $V_{N}$, $V_{E}$ are the northern and eastern components of the object’s linear velocity along the Earth’s surface, Ω is the angular velocity of the Earth’s rotation, and *R* is the Earth’s radius. (In this article, the linear velocity vector is considered to be directed along the vehicle’s longitudinal axis of symmetry in the direction of its motion; i.e., course and heading angles are considered equal). Orientation of the Résal’s system relative to the GCS, as shown in Figure 2, is determined by two angles *A* and *h*.

There is no point in presenting here the derivation of the DG’s equations of motion, which have been repeatedly obtained by a number of authors ([2,7,9] and many others). With the possible exception of designations, they all will have the following form:

(1) $$\begin{array}{cc} \dot{h}\cos h+\omega_{\xi }\cos A\cos h+\omega_{\eta }\sin A\cos h\;\;\;\; & =\;\;\omega_{p\zeta }, \end{array}$$

(2) $$\begin{array}{cc} \dot{A}\cos h+\omega_{\xi }\sin A\sin h-\omega_{\eta }\cos A\sin h+\omega_{\zeta }\cos h & =-\omega_{px}, \end{array}$$

where $\omega_{p\zeta }$ and $\omega_{px}$ are the precession angular velocities of the DG about the corresponding axes, equal to $M_{\zeta }/H$ and $M_{x}/H$; $M_{\zeta }$ and $M_{x}$ are the total torques—comprising the torques applied by the torque generators (about the axes of the outer gimbal and gyro element gimbal, respectively), as well as those caused by gimbal and gyro imperfections (gimbal bearing friction, residual rotor unbalance, etc.).

The main goal of this work is to address the issue of the selectivity of the 2dfbg-based DG with respect to the meridian line, despite the fact that in gyroscopic theory works known to the authors—for example, refs. [6,9]—the answer to this question is unequivocally negative, and has not been disputed since their publication. Moreover, the DG itself as a topic of scientific research has largely disappeared from the current agenda, due to the consensus of the scientific community on the completeness of analysis of its equations of motion.

For theoretical purposes, the proposed methodology for studying the DG stability is designed to ensure the highest possible validity of the results over a wide range of initial conditions (ICs) and other parameters—wider than the typical practical range of those values.

This analysis examines the stability of the equations of motion of a 2dfbg “on an even keel” over a long period (≫24 h):

1. in free gyroscope mode;
2. in slaved mode and with the DG in its standard operating position (position in which the angle between the gyro spin vector and the meridian line is small), with varying initial conditions and precession torques, as well as during the vehicle’s 2D maneuvering.

## 4. Results of the Study of the Equations of Motion

Analytical integration of a system of nonlinear inhomogeneous differential Equations (1) and (2) is generally difficult. For the case of zero right-hand sides ($\omega_{p\zeta }=\omega_{px}=0$, i.e., in the absence of slaving or any other external torques applied to the gimbal axes), P. I. Saidov ([9]) and A. M. Letov ([11]) obtained a solution to these equations by quadratures. Below, we study both this mode (i.e., free gyro) and the effect of slaving and other external torques; therefore, we will further seek solutions to (1), (2) using numerical integration based on an implementation of Verner’s “most robust” Runge–Kutta 9(8) pair with an 8th-order continuous extension [12]—the “ode89” function from the MATLAB^®^ v. 2022a numerical computing language.

### 4.1. 2dfbg in Free Gyro Mode

The behavior of the 2dfbg with $\omega_{p\zeta }=\omega_{px}=0$ (i.e., with zero slaving applied through the torque generators and negligible interfering torques) is fully consistent with the paradigmatic concepts of the apparent motion of a free gyro on the surface of the rotating Earth.

**Figure 3:**
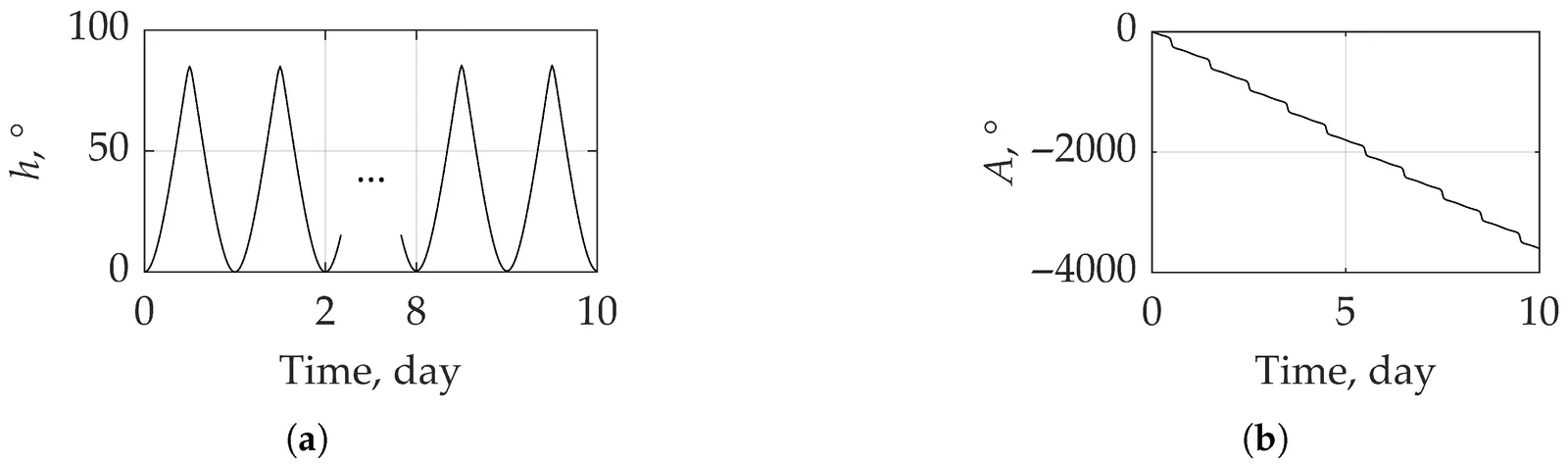
Time-dependence plots of the rotation angles of the (**a**) inner, and (**b**) outer frames.

The plots in Figure 3 correspond to the ICs $A_{0}=0{}^{\circ}$ and $h_{0}=0{}^{\circ}$ for a stationary object ($V_{N}=V_{E}=0$, just as in further simulations laid out in this section) residing at φ=47.5°.

When displaced—including by the *h* angle—from the meridian line (i.e., when the free gyro’s ICs change), it behaves similarly to that shown in Figure 3—right until the gyro spin vector intersects with the conical surface with an aperture of (180°−2φ), which is swept by the local vertical during its daily rotation around the Earth’s axis. When the gyro spin axis and the local vertical coincide (or, rather, approach each other, considering the engineering implementation), the free gyro in the Alt-Az gimbal loses its functionality (an effect known as “gimbal lock”).

**Figure 4:**
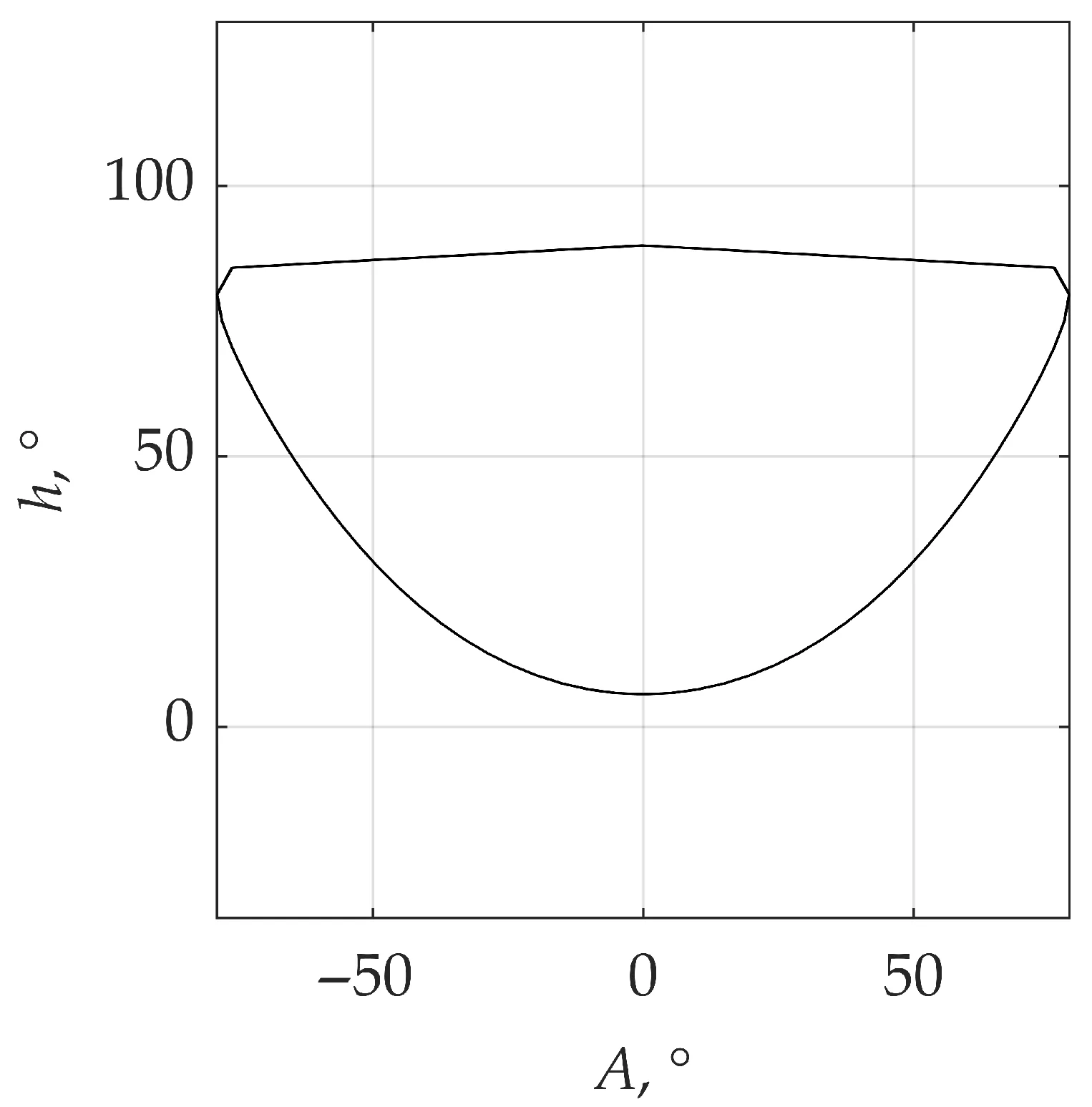
The h(A) hodograph when the gyro spin vector is within the “cone of vertical”.

**Figure 5:**
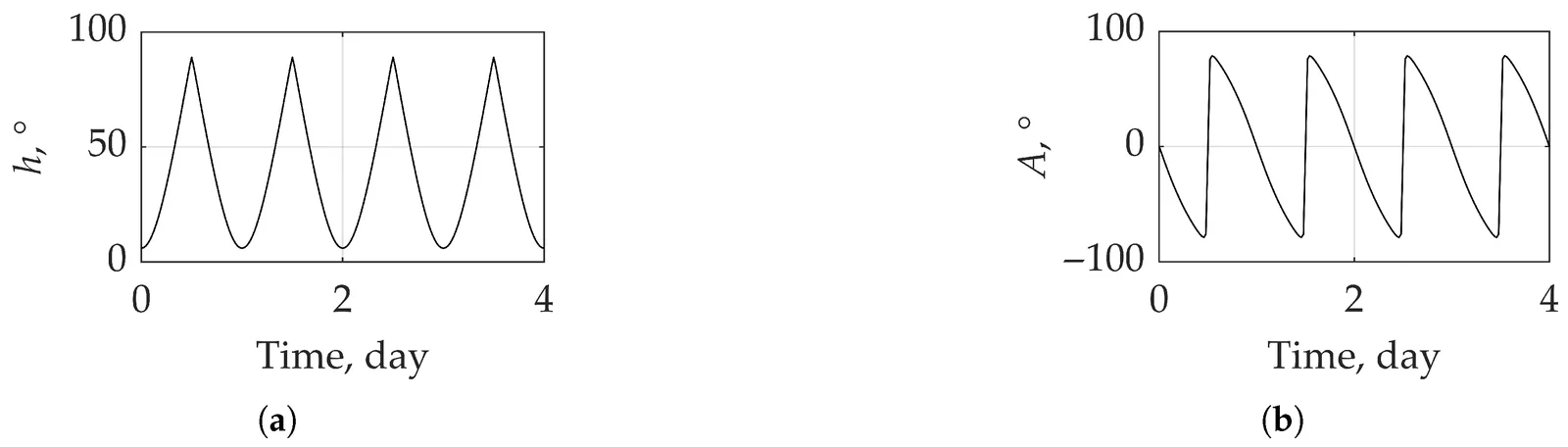
Time-dependence plots of the rotation angles of the (**a**) inner, and (**b**) outer frames near the gimbal lock zone.

However, as it moves away from the generatrix inwards the aforementioned “cone of vertical”, the gyroscope will acquire stability, illustrated in Figure 4 with a hodograph plotted in the (*h*, *A*) tangent plane. The shape of this hodograph characterizes a system possessing non-asymptotic stability [13] or being on its brink (which in this case is equivalent). In Figure 4, which corresponds to φ=47.5°, $h_{0}=6{}^{\circ}$, and $A_{0}=0{}^{\circ}$, the nature of the apparent motion of the free gyro’s spin axis is determined by its proximity to the gimbal lock zone. This is even more clearly seen in Figure 5, which shows the time plots of *A* and *h*.

Back in his day, one of the authors of this paper formulated a criterion for the free gyro’s “polarity”—a condition for maintaining its selectivity toward the celestial axis when navigating at high latitudes: the head of the rotor spin vector must be located within the aforementioned “cone of vertical”. Now we are going to generalize this criterion, by removing the high latitudes assumption.

The strict criterion for the free gyro’s “polarity”, which we herein define as non-asymptotic stability relative to the celestial axis, is expressed by a combination of two conditions: (1) both *y* and ζ axes are directed towards, and intersect, the same hemispherical surface of infinite radius, corresponding to one of latitudinal hemispheres of Earth (Northern or Southern); and (2) inequality |δ|<|φ| is satisfied (where δ is the angle between the gyro spin vector and the equatorial plane). In the early days of development of free gyros with electrostatically suspended rotors ([14]), it was common to liken them to celestial bodies, describing their coordinates in the horizontal (Darboux) and celestial (quasi-inertial) frames. This allows, among other things, the use of the following spherical trigonometry relations for calculating δ [15]:

(3) $$\left\{\begin{array}{cc} \cos\tau \cos\delta & =\cos\varphi \sin h-\sin\varphi \cos h\cos A;\\ \sin\tau \cos\delta & =-\cos h\sin A;\\ \sin\delta & =\sin\varphi \sin h+\cos\varphi \cos h\cos A, \end{array}\right.$$

where τ is the hour angle.

This approach avoids solving differential Equations (1) and (2) in the absence of slaving torques, since their integrals, which relate the free gyro’s coordinates in the inertial and geographic frames, are indeed represented by that very Equation (3); this is effectively the same aforementioned solution by Saidov and Letov, albeit in a different form. Despite that, we use numerical integration here as well—for unification purposes. The adequacy of said numerical integration in the general case is additionally confirmed by conformance between the numerical solutions to (1), (2) with zero right-hand sides, and values computed using (3), for a number of δ and φ values (10 each, i.e., 100 total comparisons). The difference turned out to be in the order of 10^−12^ degrees. As such, the curves plotted in accordance with (3) within the same axes as those shown in Figure 4 and Figure 5 are visually indistinguishable from them and are thus not depicted separately.

The “polarity criterion”, which—for the first time—was formulated above, might prove useful in choosing the optimal orientation of the free gyro in single rotor systems [16], but this is beyond the scope of this paper.

### 4.2. The Influence of Slaving on the DG’s Stability

Let us now return to Figure 3a, which, as noted above, shows the apparent wander of the free gyro from the meridian line, with which its gyro spin axis was initially aligned. It is clear that using such a gyroscope in DG mode in practice would be awfully unwieldy.

In principle, it is possible to compensate for its apparent wander by means of calculation, subtracting the integral of the vertical component of the Earth’s daily rotation angular velocity from the values of angle *A* obtained from ${\mathrm{SG}}_{1}$ (Figure 2). However, in the early days of gyro-instrument engineering, implementing even such a simple algorithm was fraught with inconveniences due to the state of computing devices of the time. As for the context of the issues explored in this paper, we also note that implementing such a “feedbackless” DG makes any talk of stability impossible. Therefore, for most of the history of the use of DGs (at least since World War II), control circuitry has already provided compensation for the object’s transport angular velocities by controlling the 2dfbg’s precession using torque generators.

Below, the stability of this mode with respect to non-zero initial conditions, errors in precession torques, and vehicle’s 2D maneuvering, is investigated.

**Figure 6:**
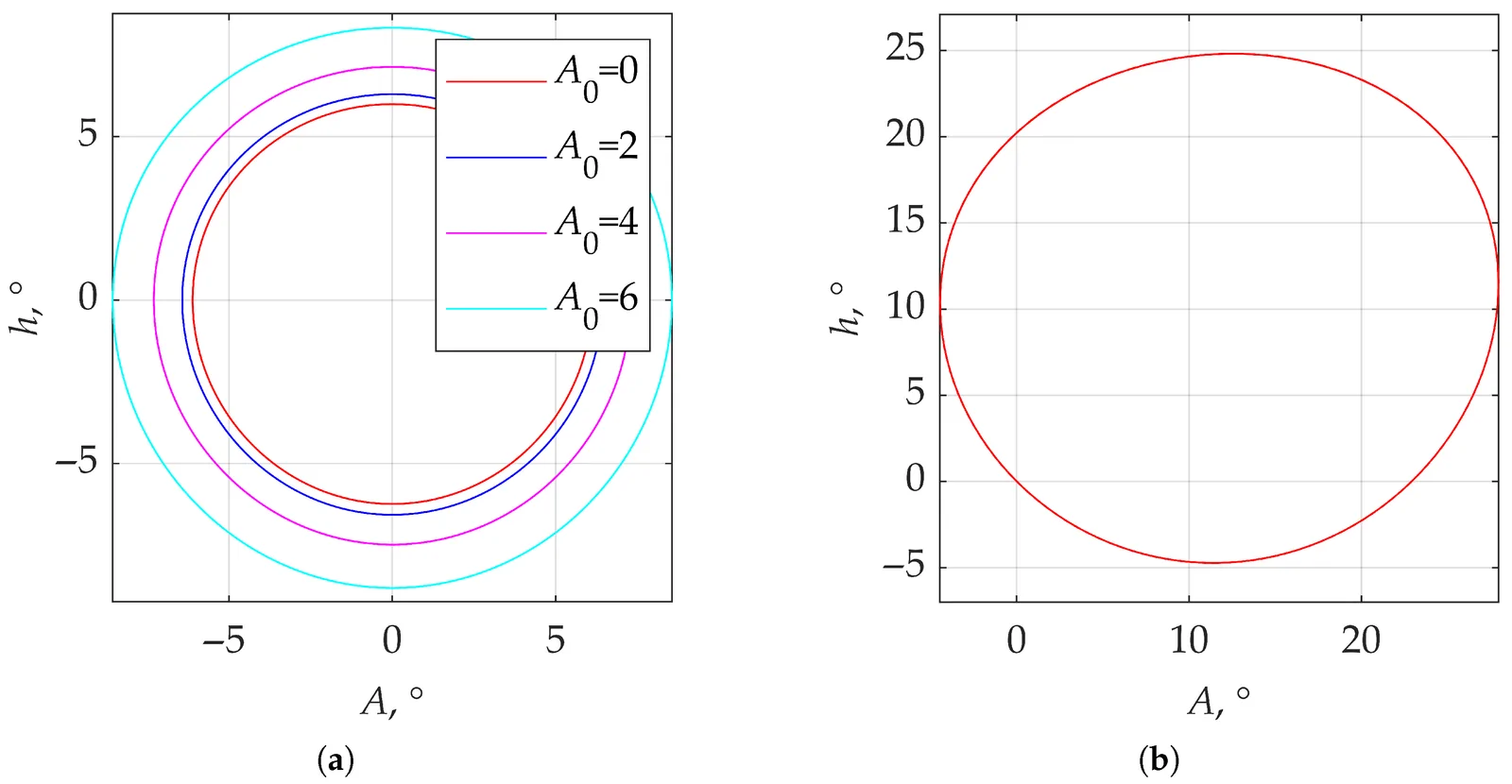
h(A) hodographs for (**a**) non-zero ICs and $\omega_{px}=-\Omega \sin\varphi$, (**b**) zero ICs and the presence of interfering torques (M/H=2 °/h).

In light of the above, we begin the analysis of the DG’s motion by introducing compensation for the vertical component of the Earth’s daily rotation angular velocity, assuming $\omega_{px}=-\omega_{\zeta }=-\Omega \sin\varphi$. We will consider (1), (2) for an ideal 2dfbg under the same conditions (φ=47.5°, $V_{N}=V_{E}=0$, $\omega_{p\zeta }=0$, $A_{0}=0{}^{\circ}$, $h_{0}=0{}^{\circ}$), for which the curves in Figure 3 were plotted. Obviously, in this case the DG will have no apparent motion, and instead will maintain the direction of the true meridian line. In the presence of certain initial deviations of the gyro spin axis from the meridian line ($A_{0}=\left(0{}^{\circ};2{}^{\circ};4{}^{\circ};6{}^{\circ}\right)$, $h_{0}=6{}^{\circ}$), the shape of the hodographs (Figure 6a) is going to indicate non-asymptotic stability of the DG.

In this case, it is easy to formulate strict criteria for acceptable deviations from the meridian line, under which the DG’s selectivity to its direction is preserved. However, whilst the practical interest in different orientations of free gyros—from “equatorial” to “polar”—is determined by the tasks and composition of the navigation system as a whole [16], for the DGs it makes no sense to consider initial deviations from the desired direction that would exceed the errors of its alignment by means of external sources—in particular, magnetic compass (single-digit degrees) and accelerometer (<0.1°). And yet—foregoing general equations—we note as an example, that at φ=47.5° and horizontal position of the free gyro’s spin axis, its selectivity will be maintained even with $A_{0}$ increasing to as high as 51°, beyond which an apparent wander in *A* will be observed, similar to Figure 3a.

Let us now address the influence of interfering torques, caused either by the slaving torques (in this case, $M_{\zeta }$) being inadequate to the required precession angular velocities, or by gimbal and gyro imperfections. (Strictly speaking, the influence of such errors should be taken into account only if the gimbal is a part of 2dfbg as opposed to an inertial platform, since the motion of the latter is only indirectly affected by the disturbance torques on the gimbal axes and is determined primarily by the error model of the specific IG type. Then again, this does not qualitatively affect the analysis results). Assuming that the interfering torques are constant as part of the conducted study of the DG’s non-asymptotic stability, it is easy to verify that said stability is preserved even when the slaving torques deviate significantly from their required values. See e.g., Figure 6b, where, for the conditions φ=47.5°, $V_{N}=V_{E}=0$, $A_{0}=0{}^{\circ}$, and $h_{0}=0{}^{\circ}$, $\omega_{px}$ and $\omega_{p\zeta }$ are introduced with errors of +2 °/h—an intentionally huge value (greater than what can be caused by possible errors of DG’s adjustment by external sources) for illustrative purposes.

It should be noted that a similar non-asymptotic stability will also be characteristic of the devices mentioned in the Introduction as “conceptually close” to the DG (“Vertikant” and “Horizont”), and in general of all those 2dfbgs whose purpose is to indicate the direction of earth-fixed reference points.

**Figure 7:**
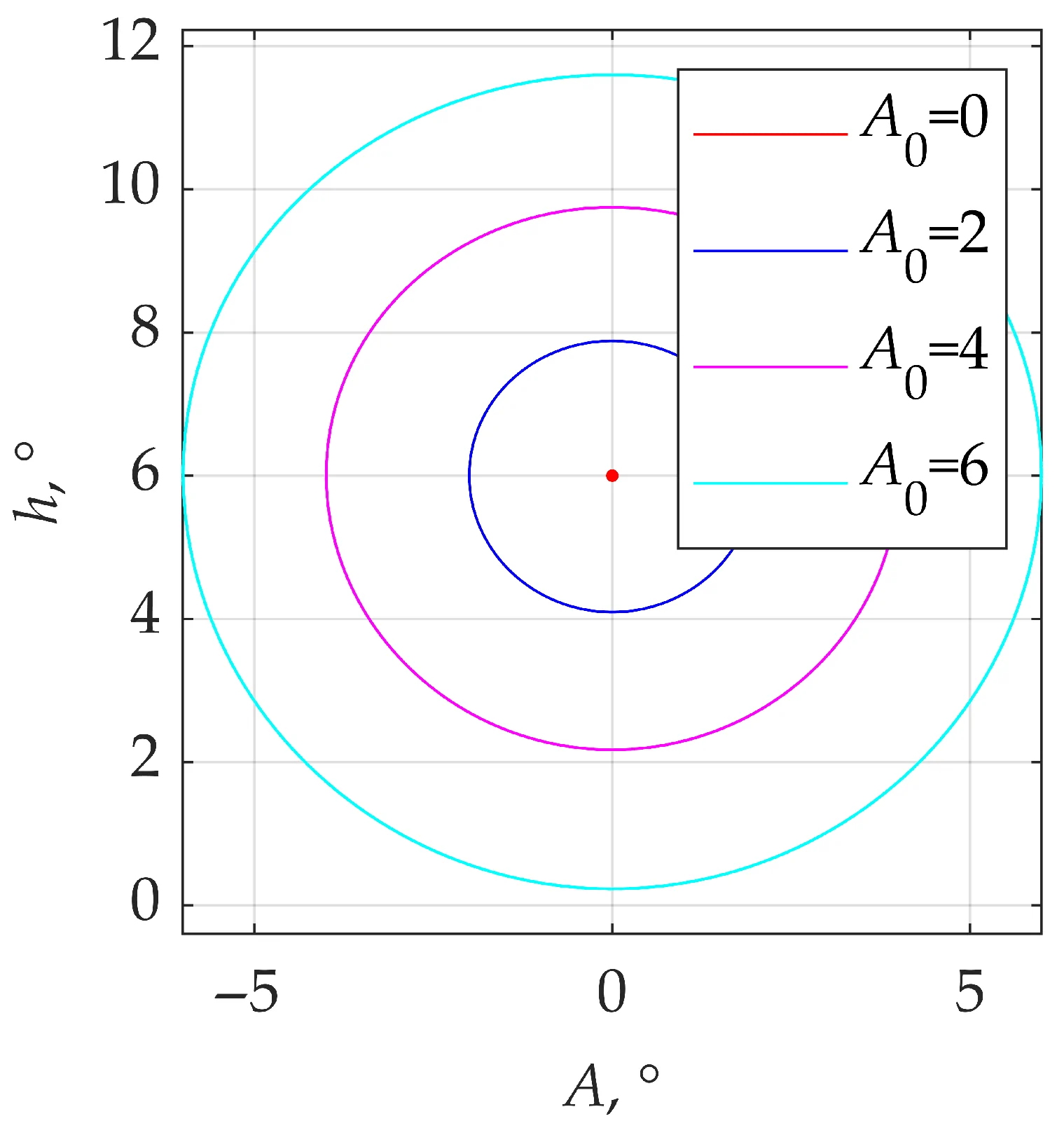
h(A) hodographs for non-zero ICs and $\omega_{px}=-\Omega \sin\left(\varphi -h_{0}\right)$.

So, for example, if the 2dfbg’s gyro spin axis has been oriented with an initial deviation of $h_{0}$, it is possible to take this value into account as part of the slaving torque by providing $\omega_{px}=-\Omega \sin\left(\varphi -h_{0}\right)$. In this case, for $A_{0}=0{}^{\circ}$ and no disturbance torques, the gyroscope will maintain its given direction (0°, $h_{0}$) with zero deviations. Under non-zero initial conditions, such compensation will also provide non-asymptotic stability. This can be seen in Figure 7 showing hodographs for $A_{0}=\left(0{}^{\circ};2{}^{\circ};4{}^{\circ};6{}^{\circ}\right)$, $h_{0}=6{}^{\circ}$, whose center has shifted to the point (0, 6).

To conclude the stability analysis of the DG, we consider the effect of vehicle’s 2D maneuvering.

**Figure 8:**
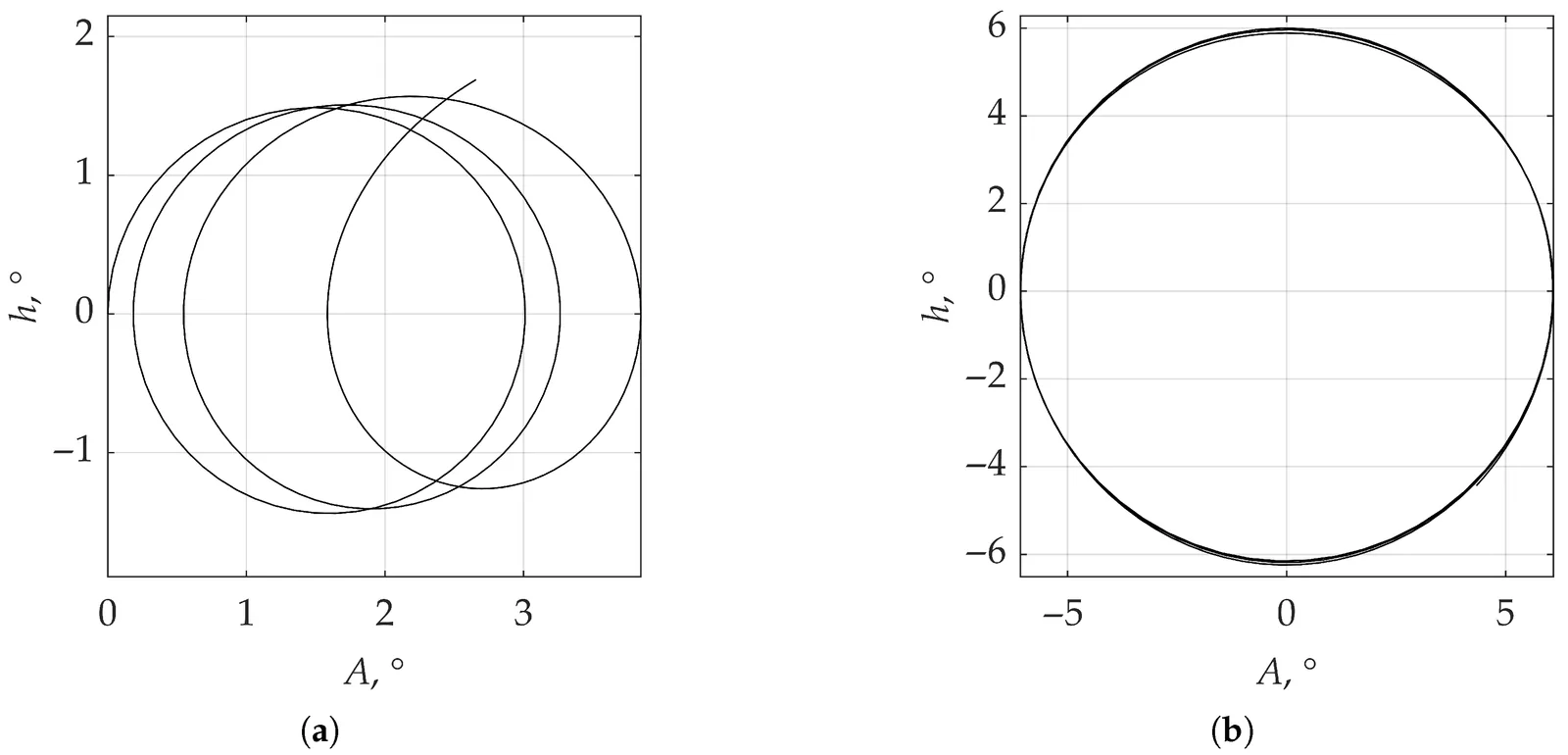
h(A) hodographs for movement along the meridian: (**a**) for $A_{0}=0{}^{\circ}$, $h_{0}=0{}^{\circ}$ and without $\omega_{p\zeta }$ slaving; (**b**) for $A_{0}=6{}^{\circ}$, $h_{0}=1{}^{\circ}$ and with $\omega_{p\zeta }$ slaving.

It will be recalled that the DG’s core function is to maintain the specified azimuthal direction during the motion of the object on which it is mounted. Figure 8a shows the h(A) hodograph obtained while moving along the meridian over the course of 5 days: $\varphi =30{}^{\circ}+\frac{V_{N}}{R}t$ (increases to 70°), $V_{N}=20$ kn, $V_{E}=0$, $A_{0}=0{}^{\circ}$, $h_{0}=0{}^{\circ}$, $\omega_{px}=-\Omega \sin\varphi$, and $\omega_{p\zeta }=0$.

Obviously, the hodograph here does not have the appearance intrinsic to a DG with selectivity; however, to restore it, it is sufficient to introduce the appropriate slaving: $\omega_{p\zeta }=\omega_{\xi }=-\frac{V_{N}}{R}$. This will lead to the DG either maintaining its zero ICs ($A_{0}=0{}^{\circ}$, $h_{0}=0{}^{\circ}$), or restoring a stable elliptical shape of the hodograph (in case of non-zero ICs). In Figure 8b said non-zero ICs have been intentionally chosen to be huge (greater than possible errors of DG’s adjustment by external sources) for illustrative purposes.

**Figure 9:**
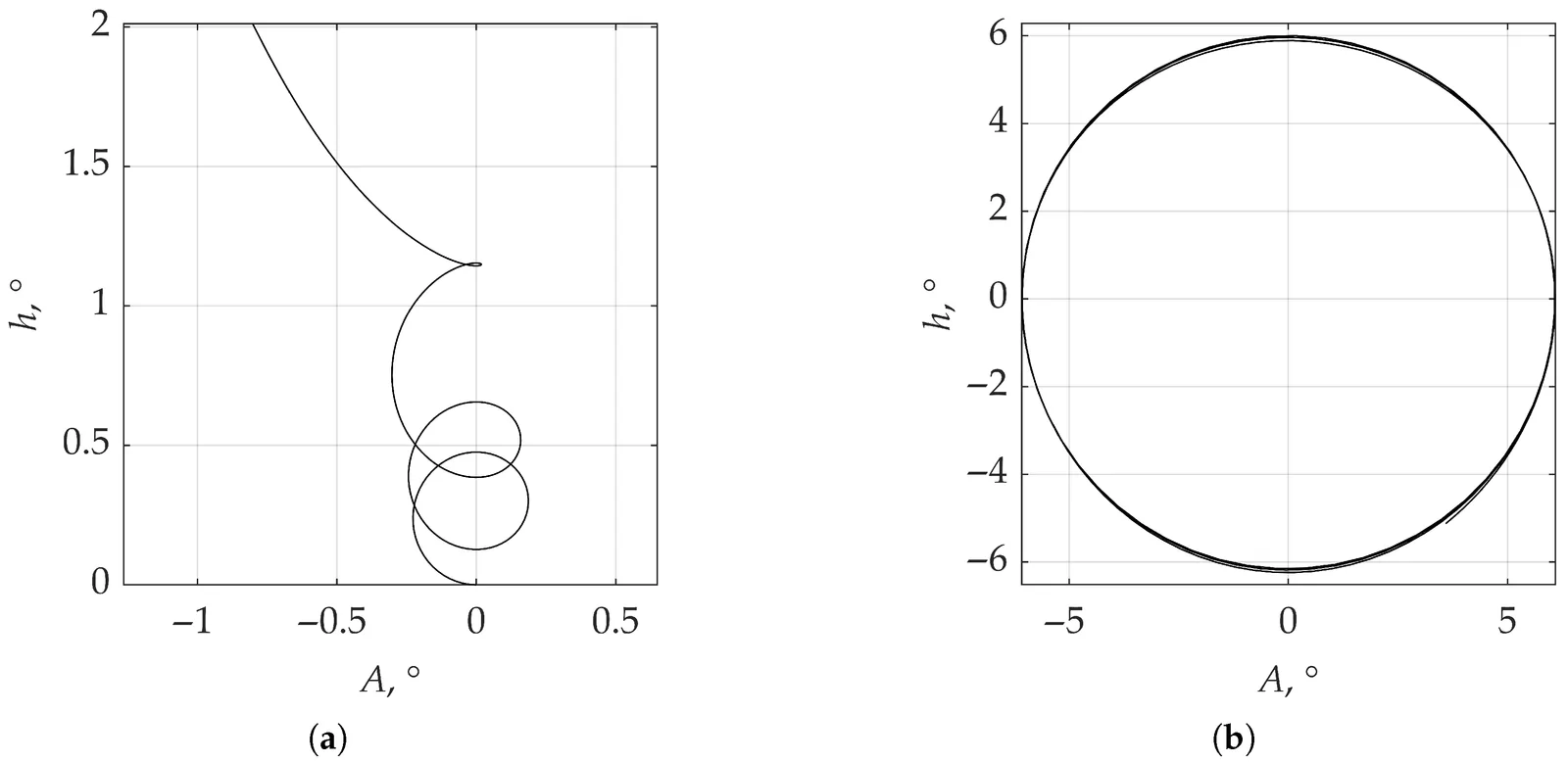
h(A) hodographs for movement with the heading of 14°: (**a**) for zero ICs and $\omega_{px}=-\Omega \sin\varphi$; (**b**) for non-zero ICs and $\omega_{px}=-\omega_{\zeta }$.

In case of arbitrary heading, an additional term should be introduced in $\omega_{px}$, bringing it into conformity with $\omega_{\zeta }$. Such a situation is shown in Figure 9, which presents the h(A) hodographs for $\varphi =30{}^{\circ}+\frac{V_{N}}{R}t$ (increases to 70°), $V_{N}=20$ kn, $V_{E}=5$ kn, $A_{0}=0{}^{\circ}$, $h_{0}=0{}^{\circ}$, $\omega_{p\zeta }=-\frac{V_{N}}{R}$ and $\omega_{px}=-\Omega \sin\varphi$ (Figure 9a); and for $\omega_{px}=-\Omega \sin\varphi -\frac{V_{E}}{R}\tan\varphi$, $A_{0}=6{}^{\circ}$, $h_{0}=1{}^{\circ}$, and all other conditions being the same (Figure 9b).

## 5. Discussion of the Results

The results presented in the previous section—which concern the DG scheme with compensation for the object’s transport angular velocity using torque generator slaving (i.e., maintaining the 2dfbg’s spin axis pointing north)—could very well have been obtained in the middle of the last century. One can only assume ([11]) that—as in the case of the free gyro discussed above—the then-current level of 2dfbg’s instrumental errors did not allow for serious consideration of long intervals of autonomous DG operation. However, even in the later (1970s–1980s) period of the development and implementation of externally corrected gyrocompasses, the hypothetical selectivity of the DG (which is a full-fledged operating mode of an IG-controlled inertial platform) did not become a subject of research. This is likely explained by the general shift in interest to the rapidly developing inertial navigation systems, believed to solve all issues of orientation and navigation. That said, we shall leave the issues of retrospective analysis to researchers of the history of gyroscopic navigation (e.g., [17]).

The non-asymptotic stability of the standard DG scheme within operational range significantly exceeding the practical errors of initial alignment, external navigation information, and precession torques, is confirmed by the results of the numerical solution of (1), (2) obtained in the previous section. As for the realistic range of deviations, these equations can be easily linearized with the usual small-angle approximations (cosA=cosh=1, sinA=A, sinh=h) and represented as:

(4) $$\begin{array}{ccc} \dot{h}+\omega_{\eta }A=\; & \omega_{p\zeta }+\frac{V_{N}}{R}, & \end{array}$$

(5) $$\begin{array}{cc} \dot{A}-\omega_{\eta }h=- & \omega_{px}-\frac{V_{E}}{R}\tan\varphi -\Omega \sin\varphi . \end{array}$$

Obviously, for $V_{E}\ll R\Omega \cos\varphi$, the differential equations describe a stationary system—a harmonic oscillator with a natural frequency of $\omega_{\eta }$. Their integration by quadratures for the same $A_{0}$ and $h_{0}$ values as the numerical solution of the original (1) and (2) (Figure 6a) yields very similar results. The difference between them predictably increases with the initial misalignment between the 2dfbg and the meridian line, and also tends to increase over time.

The study of the DG’s accuracy is not the purpose of this paper; therefore, we shall not overload it with a rather cumbersome comparative analysis of the error equations for obtaining the meridian line position, which can be acquired by variation of (1), (2) and (4), (5). Its result is easily verified and quite expected: even for rather large (up to 5°) values of $A_{0}$ and $h_{0}$, which determine the trajectory of the DG’s spin vector, the residuals of the solutions for both systems of error equations are insignificant. While outlining the main properties of the DG, we will work with the linearized trajectory Equations (4) and (5). They clearly demonstrate that the equilibrium azimuthal position of the estimated meridian line will (just as in the gyrocompass case) be determined by the errors in (a) the vehicle’s measured northern velocity, and (b) compensation for the projections of the 2dfbg’s wander onto the eastern axis of the GCS. As for the position of the estimated meridian line relative to the horizontal plane, it is significantly different for the DG from that for the gyrocompass; this may be of interest for research beyond the scope of this paper.

The physical interpretation of this meridian selectivity has nothing to do with pendulosity, which has not yet been discussed. A completely different effect is at work here, which can be likened to “autocompensation” [18]. It is inherent to 2dfbgs whose gyro spin axis—due to its orientation (“polar” free gyro), and/or due to slaving torques compensating for apparent motion within the Earth-fixed frame (e.g., DG)—occupies a constant position relative to them, while at the same time not belonging to the equatorial plane.

Naturally, the gimbal kinematics also remains a limitation. In particular, the Alt-Az gimbal considered in the article, due to its “gimbal lock” effect, does not allow determining the direction of the local vertical (at least not without some sort of pendulum). This would require a different type of gimbal with two nominally horizontal axes; see, for example, refs. [6,9]. In this case, the natural frequency of oscillations around the vertical is equal to Ωsinφ. In turn, such a gimbal, in general, does not allow obtaining the position of the meridian line. Let us end this brief discussion of the gimbal kinematics role with a reminder that a universal solution for the DG and conceptually similar devices is the introduction of a third gimbal frame (see, for example, [2,6]).

Returning to the physical interpretation of selectivity and once again emphasizing that it is equally characteristic of the DG and gyrocompass, let us ask: what properties can the DG acquire when slaved to a pendulum (accelerometer)? This question should be considered within the framework of the aforementioned dual-mode externally corrected DG/gyrocompass scheme. It implies the following (not mutually exclusive) answers:

(a) Pendulosity is introduced as a g·h signal from the corresponding accelerometer with a certain coefficient *Q* into Equation (5), transforming it into (6). (Questions related to the choice of the *Q* coefficient (“Schuler tuning”), as well as the inner workings of ballistic deflections and methods for countering them, are not considered in this paper). The latter, together with the unchanged Equation (4), describes another harmonic oscillator, with a natural frequency differing from the original by a factor of $\frac{Qg}{\Omega \cos\varphi }+1$ (which can amount to dozens or hundreds of times). Its hodograph is a very flattened ellipse (deformed from a circle in the DG case), with the ratio of the semi-axes being equal to the aforementioned factor. This is nothing other than the gyrocompass—with its advantages and limitations, described in numerous works. Said limitations include the same loss of selectivity (and, essentially, performance) as φ approaches 90°, as that of a feedbackless DG. In this case, when setting the pendulosity coefficient *Q* to −Ωcosφ, the *A* value becomes an integral of the right-hand side of (5), which corresponds to the operating mode in transverse-pole spherical coordinates [19].

(b) Pendulosity allows for DG (and gyrocompass) damping, i.e., converting the non-asymptotic stability of the DG into asymptotic one. To achieve this, the same g·h signal is introduced into the left-hand side of Equation (4) with coefficient *N*, transforming it into (7). The *N* values required to achieve the same damping efficiency for the gyrocompass and DG are understandably very different—in fact, their ratio is the same as the ratio of the aforementioned natural frequencies.

(6) $$\begin{array}{cc} \dot{A}-\omega_{\eta }h-Qgh=- & \omega_{px}-\frac{V_{E}}{R}\tan\varphi -\Omega \sin\varphi , \end{array}$$

(7) $$\begin{array}{ccc} \dot{h}+\omega_{\eta }A+Ngh=\; & \omega_{p\zeta }+\frac{V_{N}}{R}. & \end{array}$$

In concluding the discussion of the obtained results, it is worthwhile to attempt to give them a certain place within the systematized body of knowledge on gyroscopic navigation and orientation devices. Perhaps filling a theoretical gap—even on a problem that has seemingly lost its relevance—is justifiable on its own. That being said, experience suggests that such cases often generate new scientific and methodological interpretations of rather relevant systems, and sometimes even new technical solutions for them. Within the scope of the DG scheme (the viability of which is declared in this paper), an example of such relevant challenge is maintaining a stable (“non-wandering”) direction within an Earth-fixed frame. Inertial configurations used for determining orientation angles as a part of an inertial navigation system do not possess this property: neither the GAVVM (gyroscopic angular velocity vector meter—a vehicle-fixed triad of angular velocity sensors), nor the IG-based inertial platform are capable of directly obtaining a stable direction of the meridian line without using information from a pendulum of some kind. This allows us to expect some new properties to be achieved in a DG-based orientation system. However, it must be acknowledged that today the overwhelming majority of gyroscopic orientation systems are implemented based on angular velocity sensors. Therefore, attempts to design a composite system based on said angular velocity sensors which would combine the SINS and gyrocompass algorithms can only be welcomed [20].

The authors plan to continue further development of the topic discussed in this article.

## 6. Conclusions

This paper demonstrates that the trajectory of a classic directional gyroscope in an altazimuth gimbal is, in the general case, non-asymptotically stable and represents a circle formed by continuous quadrature harmonic oscillations around an equilibrium position—the meridian line—with frequency equal to the horizontal component of Earth’s daily rotation angular velocity. A single-rotor pendulum gyrocompass, which has a similar kinematic design, has the same equilibrium position. There, an elliptical motion around the meridian line is formed by quadrature oscillations with drastically different amplitudes. The frequency of these oscillations—e.g., Schuler’s frequency—differs from the horizontal component of the Earth’s daily rotation angular velocity to the same extent.

It can be concluded that both the directional gyroscope and the gyrocompass are equally selective to the meridian line.

These exact patterns are inherent and even more obvious for an externally corrected dual-mode gyrocompass/gyro azimuth, based on an IG-controlled dual-axis inertial platform. Such a platform maintains the same equilibrium position—the meridian line—regardless of whether the pendulosity (based on accelerometers mounted on the platform) is switched on or off. The steady-state errors that are caused by gyro wander and errors in compensating for the projections of the transport angular velocity (caused by the Earth’s daily rotation and the vehicle’s motion along its curved surface) will also be identical. The ratio between the frequencies of rotation of the platform’s stabilized axis around the meridian line along the flattened elliptical (in the gyrocompass mode) and circular (in the gyro azimuth mode) trajectories corresponds to the ratio of the semi-axes of the said ellipse—just in the same way as for a classic gimbal-mounted rotor gyro.

## Data Availability

Computational models used in this study can be provided upon request.
